# Clinicopathological Profile and Management of Right Iliac Fossa Mass: A Retrospective Observational Study

**DOI:** 10.7759/cureus.106916

**Published:** 2026-04-12

**Authors:** Aditya T Valavan, Girish Bakhshi, Chandrakant Sabale, Yashika Gupta, Ranjitha R Maiyya, Shivangi Tiwari, Amol Kudal, Rupali P Choradia

**Affiliations:** 1 General Surgery, Grant Government Medical College, Mumbai, IND

**Keywords:** appendicular abscess, appendicular lump, ileocecal tuberculosis, psoas abscess, right iliac fossa mass

## Abstract

Introduction: Right iliac fossa (RIF) mass is a common surgical presentation with diverse etiologies, including inflammatory, infective, and neoplastic conditions. Early diagnosis and appropriate management are essential to optimize outcomes, especially in developing countries.

Methods: This retrospective observational study was conducted at a tertiary healthcare center. A total of 26 patients presenting with RIF mass were included. All patients underwent detailed clinical evaluation, laboratory investigations, and imaging studies. Management strategies were tailored according to the underlying diagnosis and clinical condition.

Results: Appendicular pathology was the most common cause, accounting for 9 of 26 (34.6%) cases. Appendicular abscess was the second most common etiology, observed in 7 (26.9%) patients. Ileocecal tuberculosis constituted a significant proportion, observed in 6 (23.1%) patients, reflecting its continued prevalence in India. The majority of patients with appendicular lump and ileocecal tuberculosis were managed conservatively. Image-guided drainage was performed in selected cases of abscess, while surgical intervention was reserved for complicated cases and suspected malignancies.

Conclusion: Appendicular pathology remains the leading cause of RIF mass. Conservative management is effective in most cases of appendicular lump and ileocecal tuberculosis, while image-guided drainage or surgical intervention is required in patients with abscess, recurrence, or complications. An early, etiology-based, individualized approach is essential for optimal outcomes.

## Introduction

A mass in the right iliac fossa (RIF) is a common clinical presentation encountered in general surgical practice and often poses a diagnostic challenge due to its wide range of etiologies. The underlying causes may involve structures such as the appendix, cecum, terminal ileum, mesentery, retroperitoneum, and occasionally pelvic organs [1]. These conditions may range from benign inflammatory processes to infective diseases and malignant lesions, making accurate diagnosis essential for appropriate management.

Appendicular pathology remains the most frequent cause of RIF mass and accounts for nearly 60-70% of cases reported in previous studies [2]. An appendicular mass develops in approximately 2-6% of patients with acute appendicitis, while appendicular abscess formation occurs in nearly 1-4% of cases [3]. Other important causes of RIF mass include ileocecal tuberculosis, carcinoma of the cecum, Crohn’s disease, mesenteric lymphadenitis, and psoas abscess [1].

The etiological distribution of RIF masses varies according to geographic location and socioeconomic conditions. In developing countries such as India, abdominal tuberculosis remains a significant cause of RIF mass, with the ileocecal region being the most commonly affected site of gastrointestinal tuberculosis. Malignant lesions, particularly carcinoma of the cecum, are more frequently observed among elderly patients [1].

Advances in imaging modalities such as ultrasonography and contrast-enhanced CT have significantly improved diagnostic accuracy. However, careful clinical evaluation continues to play an important role in the early identification and management of patients presenting with RIF mass.

Objectives

The objectives of this study are to evaluate the etiological spectrum of patients presenting with RIF mass, to analyze the clinical presentation and role of imaging modalities in diagnosis, and to assess the management strategies and outcomes in patients with RIF mass.

## Materials and methods

Study design

This was a retrospective observational study conducted at a tertiary healthcare center. As the study design was retrospective and observational in nature, no prior sample size calculation was performed. All patients presenting with a RIF mass during the study period were screened through review of medical records.

Approximately 80-90 patients were identified during the initial screening. However, only 26 patients were included in the final analysis, as inclusion was restricted to those with complete medical records, documented clinical details, and completion of treatment at our institution.

Study period

The study was conducted over a one-year period from December 2024 to December 2025.

Inclusion and exclusion criteria

Inclusion Criteria

Patients of either sex and all age groups with a documented palpable RIF mass on clinical examination or a localized mass detected on imaging modalities such as ultrasonography or CT were included.

Exclusion Criteria

Patients with masses of definite gynecological origin (e.g., tubo-ovarian cysts or ovarian neoplasms), lesions arising from other abdominal quadrants extending into the RIF, and those with incomplete medical records were excluded.

Data collection

Demographic data, including age and sex, were obtained from hospital records. Clinical history, including presenting complaints such as abdominal pain, fever, vomiting, weight loss, and abdominal lump, was extracted from case files. Relevant past history, including tuberculosis, prior abdominal surgery, or malignancy, was also recorded. Clinical examination findings and laboratory investigations -- including complete blood count, inflammatory markers, renal function tests, and liver function tests -- were retrieved from patient records. Ultrasonography findings were available for all patients, while contrast-enhanced CT findings were recorded where applicable. For patients who underwent surgical intervention, operative notes were reviewed, and histopathological examination reports of resected specimens were analyzed to establish the final diagnosis.

Statistical analysis

Data from 26 patients were summarized using descriptive statistics, including frequencies and percentages. Statistical analysis was performed using Microsoft Excel 2021 (Microsoft Corporation, Redmond, WA, USA) and IBM SPSS Statistics for Windows, version 26.0 (IBM Corp., Armonk, NY, USA).

Additional note

Language editing and grammatical refinement were assisted using OpenAI’s ChatGPT; however, all data analysis and interpretation were independently performed by the authors.

## Results

Appendicular pathology constituted the majority of cases in the present study, with appendicular lump and appendicular abscess accounting for most of the RIF masses. Infective conditions such as ileocecal tuberculosis and psoas abscess were also significant contributors, while malignancy was relatively uncommon (Table 1).

**Table 1:** Etiological distribution of right iliac fossa mass

Etiology	Number of cases	Percentage
Appendicular lump	9	34.6%
Appendicular abscess	7	26.9%
Ileocecal tuberculosis	6	23.1%
Psoas abscess	2	7.7%
Malignancy	2	7.7%
Total	26	100%

Among the 26 patients included in the study, females slightly outnumbered males. Appendicular lump demonstrated a slight male predominance, while appendicular abscess was more frequently observed among female patients. Ileocecal tuberculosis showed equal distribution between the sexes (Table 2).

**Table 2:** Sex distribution according to etiology

Etiology	Male	Female	Total
Appendicular lump	5	4	9
Appendicular abscess	1	6	7
Ileocecal tuberculosis	3	3	6
Psoas abscess	1	1	2
Malignancy	1	1	2
Total	11	15	26

The majority of patients belonged to the third decade of life, followed by the fourth decade. Appendicular pathology was more common among younger individuals, whereas malignancy occurred in an elderly patient (Table 3).

**Table 3:** Age distribution according to etiology

Etiology	11-20	21-30	31-40	41-50		51-60	Above 60
Appendicular lump	1	4	2	2		0	0
Appendicular abscess	4	2	0	1		0	0
Ileocecal tuberculosis	1	1	2	1		1	0
Psoas abscess	0	1	1	0		0	0
Malignancy	0	0	0	1		0	1

Abdominal pain and a palpable abdominal lump were the most common presenting complaints. Fever was frequently associated with inflammatory conditions such as appendicular abscess, while weight loss was more commonly observed in ileocecal tuberculosis and malignancy (Table 4).

**Table 4:** Presenting complaints according to etiology

Etiology	Pain	Lump	Fever	Vomiting	Weight loss
Appendicular lump	9	9	7	7	1
Appendicular abscess	7	6	7	5	1
Ileocecal tuberculosis	4	5	3	2	4
Psoas abscess	2	1	2	1	1
Malignancy	0	2	0	0	2

Contrast-enhanced CT (CECT) abdomen emerged as the predominant imaging modality across most pathologies, particularly in appendicular abscess, ileocecal tuberculosis, and malignancy. Ultrasonography served as the primary diagnostic modality in all cases of appendicular lump, highlighting its utility as an initial screening tool. Colonoscopy played an important role in the diagnosis of ileocecal tuberculosis and malignancy by enabling direct visualization and tissue diagnosis. PET scan contributed significantly to the evaluation of malignancy, especially in advanced cases (Table 5).

**Table 5:** Distribution of diagnostic modalities across etiologies

Etiology	USG	CECT	Colonoscopy	PET scan
Appendicular lump	9	9	0	0
Appendicular abscess	7	7	0	0
Ileocecal tuberculosis	0	6	2	0
Psoas abscess	2	0	0	0
Malignancy	0	2	1	1

Appendicular lump

**Figure 1:**
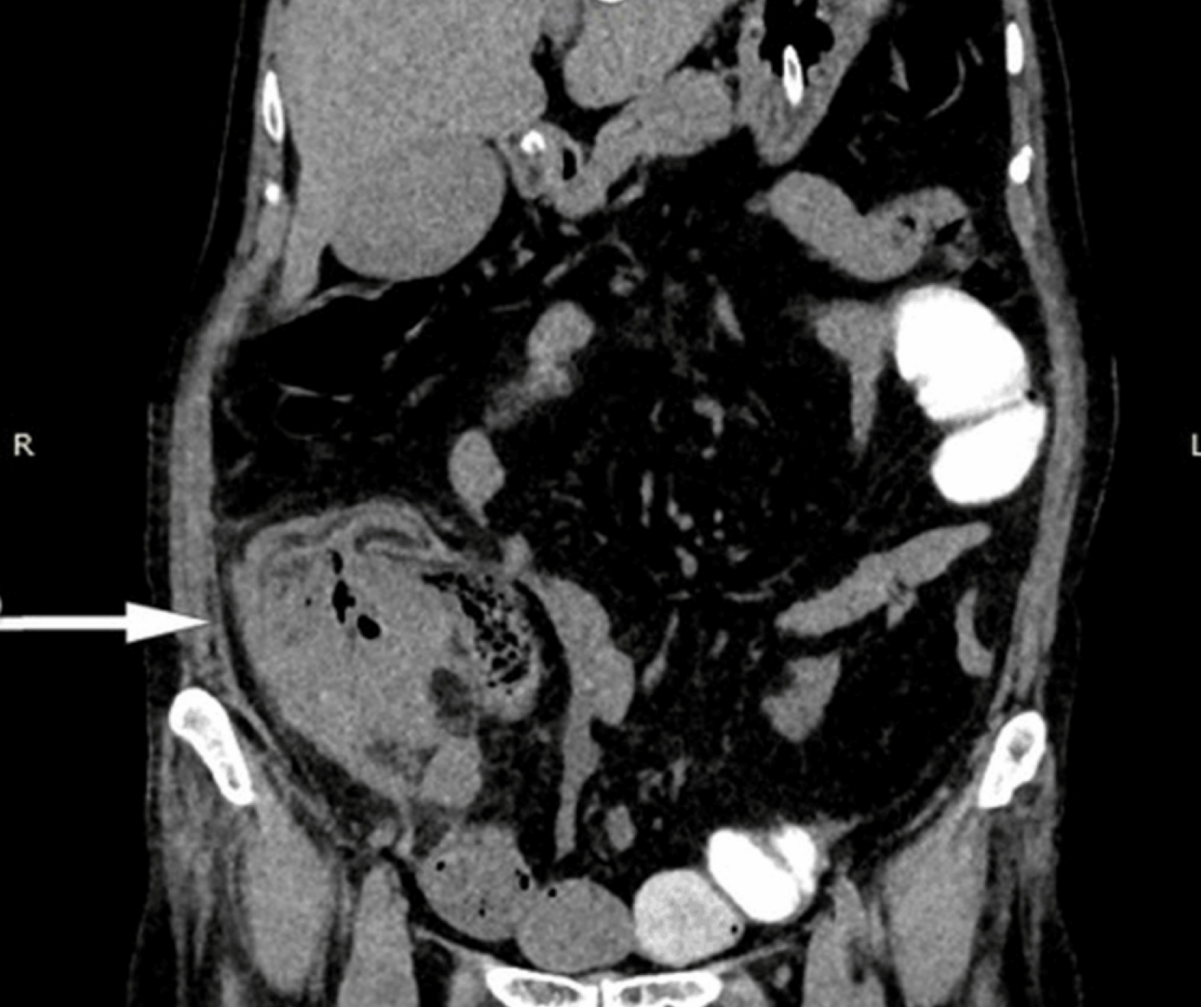
Contrast-enhanced CT showing appendicular lump formation following base of appendix perforation.

**Figure 2:**
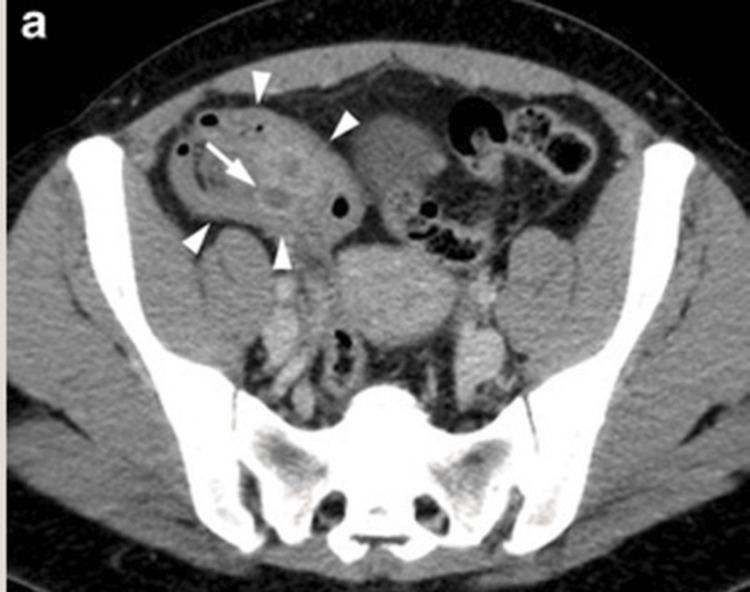
Contrast-enhanced CT showing appendicular lump (phlegmon) formation in a case of acute appendicitis.

Among the 26 patients, appendicular lump was the most common etiology, observed in nine cases, typically resulting from inflammatory mass formation secondary to acute appendicitis or sealed perforation (Figures 1, 2).

Most patients were managed conservatively with intravenous antibiotics and supportive care. In our center, initial antibiotic therapy typically consists of intravenous ceftriaxone administered for five to seven days, depending on the patient’s clinical condition, including fever, total leukocyte count, and inflammatory markers such as ESR and CRP. In cases where there is inadequate clinical response, such as persistent fever or rising inflammatory markers, antibiotic therapy is escalated to piperacillin-tazobactam. Once clinical improvement is observed, including resolution of fever for 48-72 hours and a declining trend in leukocyte count and inflammatory markers, patients are transitioned to oral antibiotics, such as cefixime, to complete the course of treatment.

**Figure 3:**
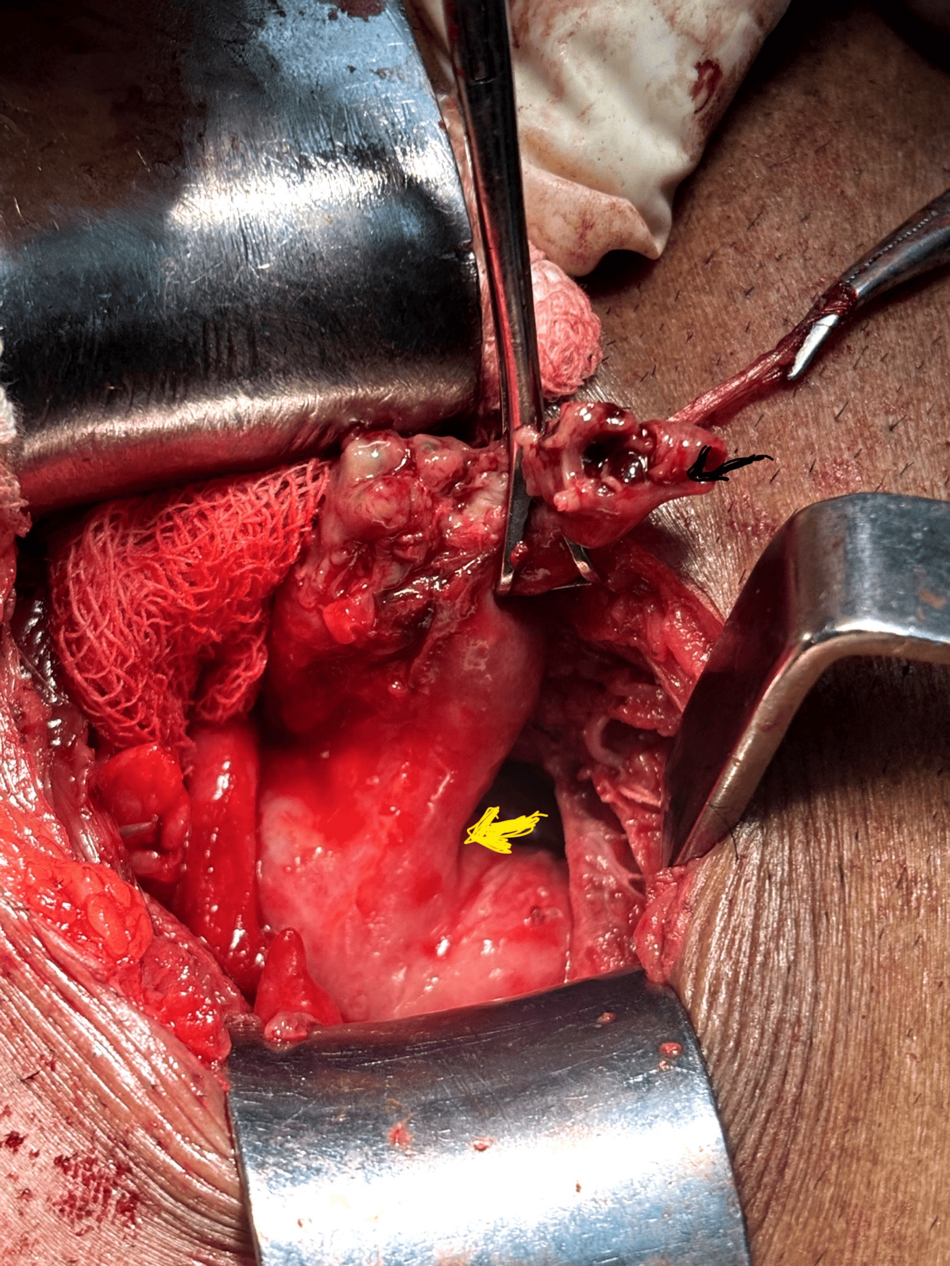
Sealed perforation of the appendix The image shows a perforated appendix (black arrow). The yellow arrow indicates the ileocecal junction. The procedure was initially attempted laparoscopically and subsequently converted to open surgery due to intraoperative difficulty.

A subset of patients developed recurrent symptoms requiring interval appendectomy, performed either laparoscopically or via open approach in the presence of dense adhesions (Figure 3). In one patient with persistent symptoms and unresolved phlegmon, further evaluation with colonoscopy and biopsy revealed ileocecal tuberculosis, and anti-tubercular therapy (ATT) was initiated.

Appendicular abscess

**Figure 4:**
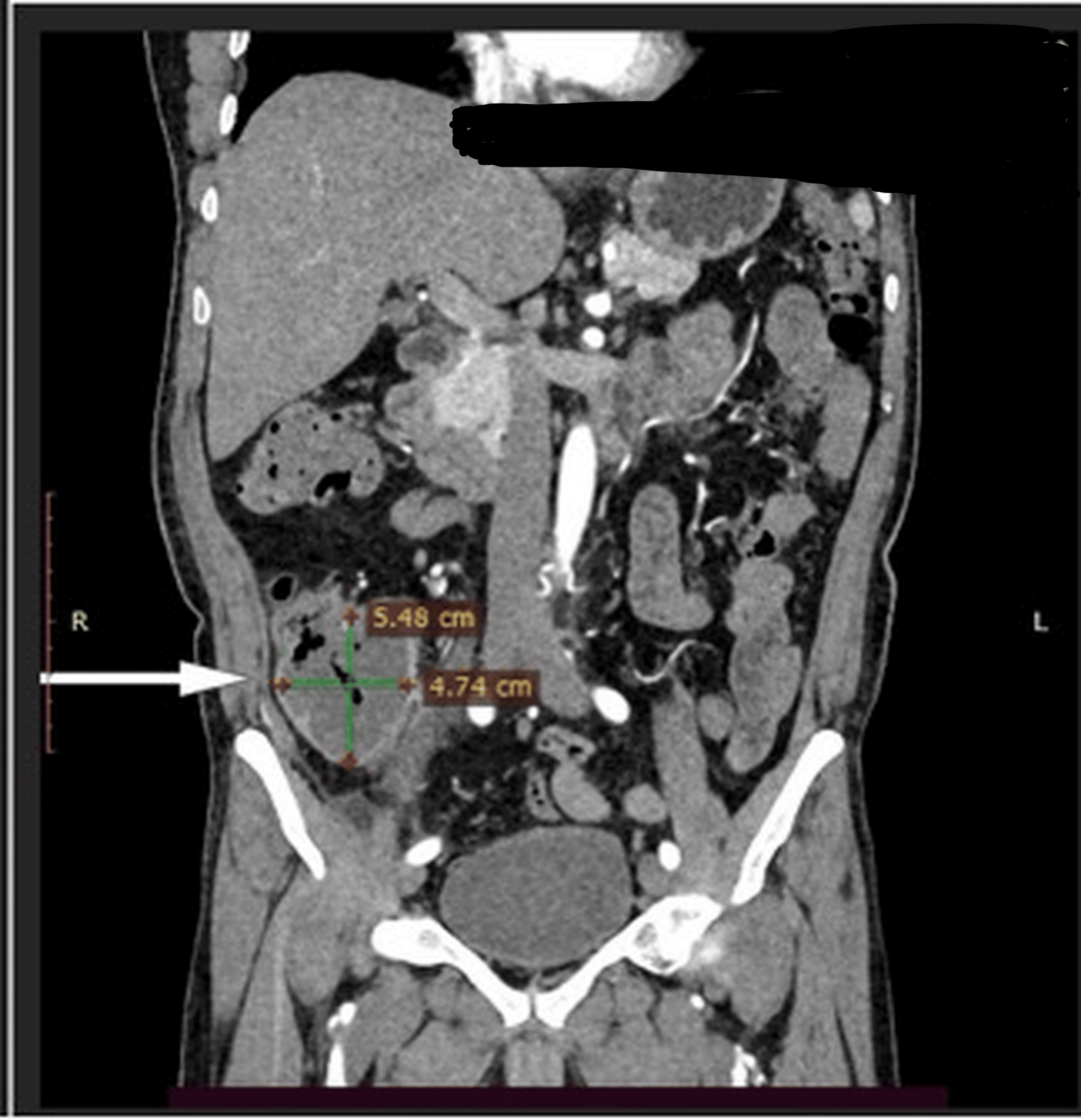
Contrast-enhanced CT abdomen suggestive of a necrotic collection of approximately 40 to 50 cc at the base of appendix

In our series of 26 patients, seven patients were diagnosed with appendicular abscess (Figure 4). All patients were initially managed with conservative treatment, including intravenous antibiotics, fluid resuscitation, and close clinical monitoring.

In our study, all patients with appendicular abscess were initially managed conservatively with antibiotics. Further management was guided by abscess size and clinical status at our center. Collections measuring less than 60 cc were managed with image-guided aspiration, while larger collections (>60 cc) were managed with percutaneous drainage (pigtail catheter). 

**Figure 5:**
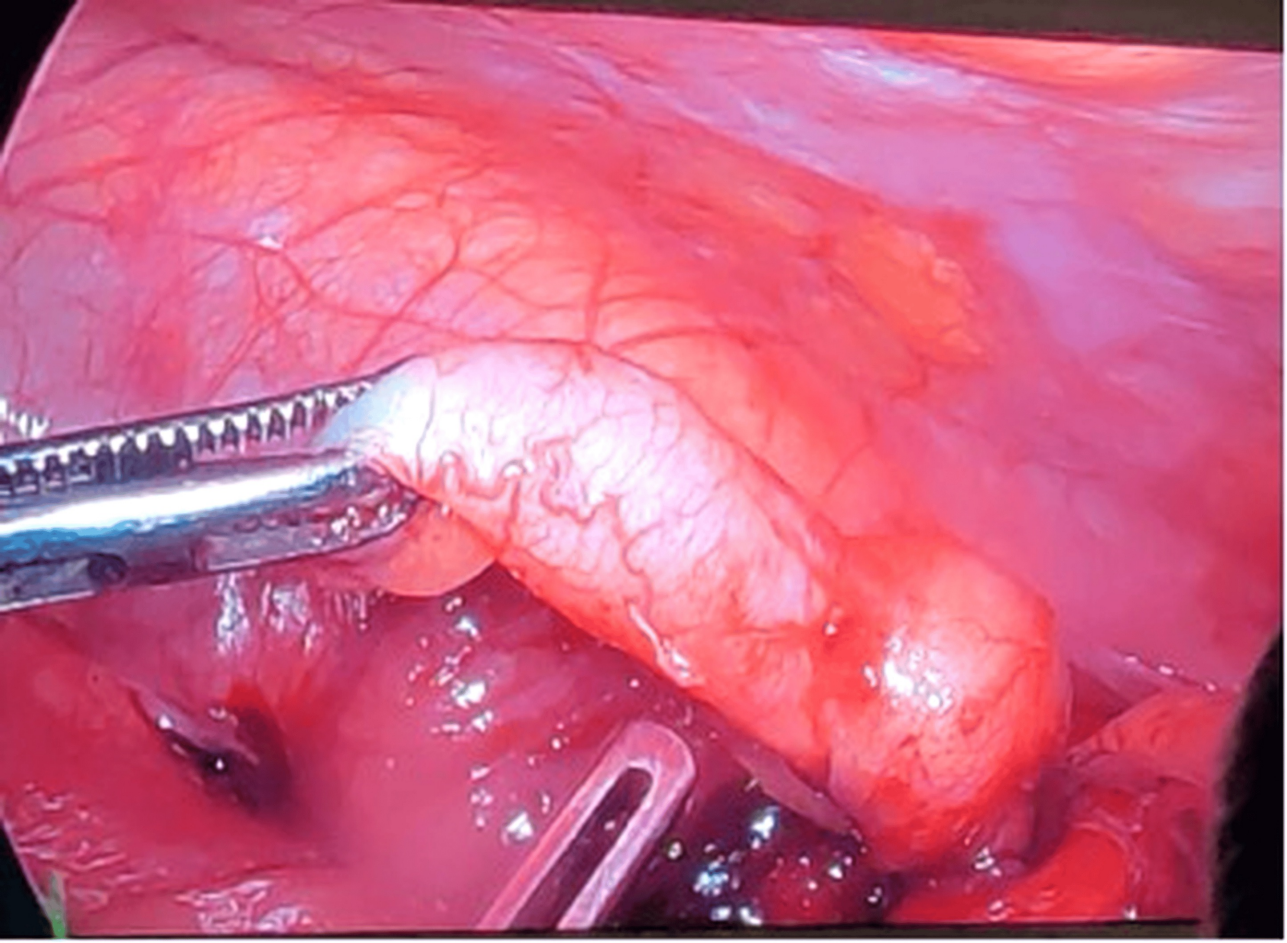
Interval laproscopic appendectomy

The case shown in Figure 4, with an estimated collection of 40-60 cc, was managed with aspiration. Among these seven patients, three experienced recurrent symptoms requiring subsequent readmission. These patients underwent interval laparoscopic appendectomy approximately six weeks after the initial presentation, once the acute inflammatory phase had resolved (Figure 5).

Ileocecal tuberculosis

**Figure 6:**
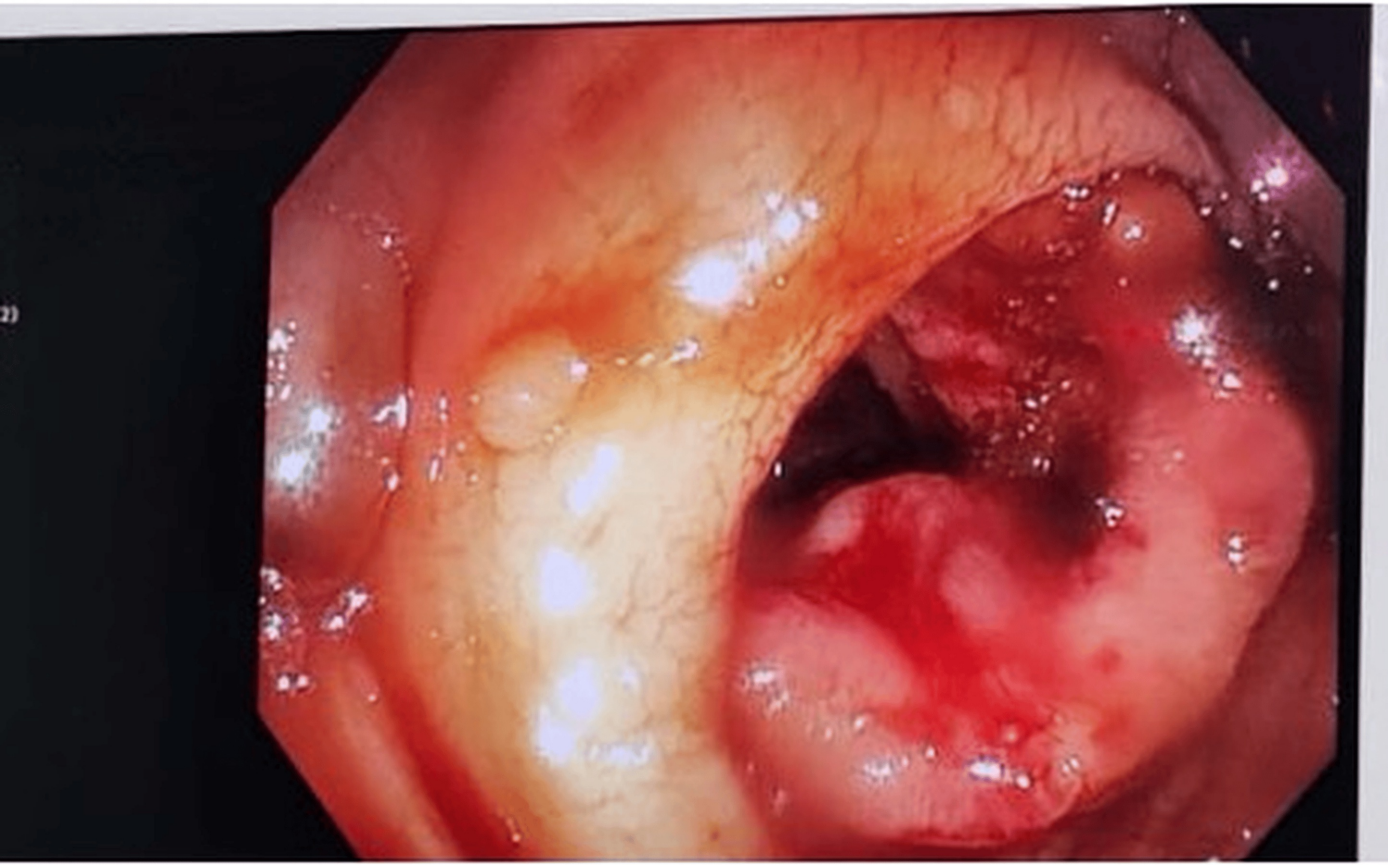
Colonoscopy-guided biopsy suggestive of abdominal Koch’s

Out of 26 patients, 6 were diagnosed with ileocecal tuberculosis, highlighting its continued prevalence and the persistent burden of tuberculosis in developing countries such as India. The majority were managed conservatively with ATT for 6 months, extended to 9 to 12 months, ultimately resulting in resolution of the disease. Colonoscopy-guided biopsy also played an important role in establishing the diagnosis and guiding management (Figure 6).

**Figure 7:**
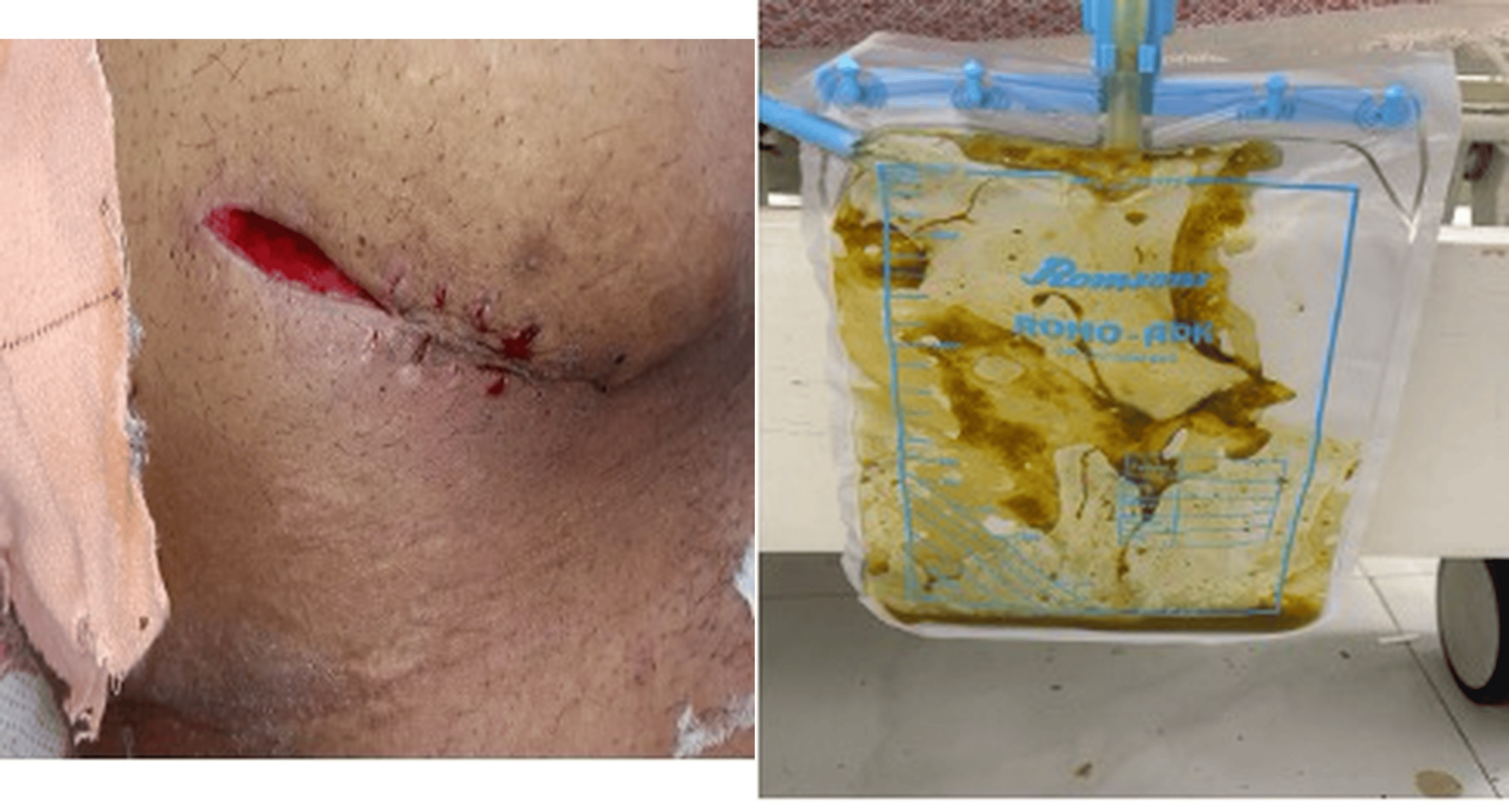
:Enterocutaneous fistula in a case of abdominal Koch’s The patient with an enterocutaneous fistula had undergone prior surgery at another center for suspected appendicitis. During intraoperative exploration, an appendicular lump was identified, and the procedure was abandoned with placement of an intra-abdominal drain. In the postoperative period, the patient developed feculent discharge through the drain and surgical site. Samples sent for analysis were positive for tuberculosis, following which ATT was initiated, and the patient was referred to our institution. ATT, anti-tubercular therapy.

**Figure 8:**
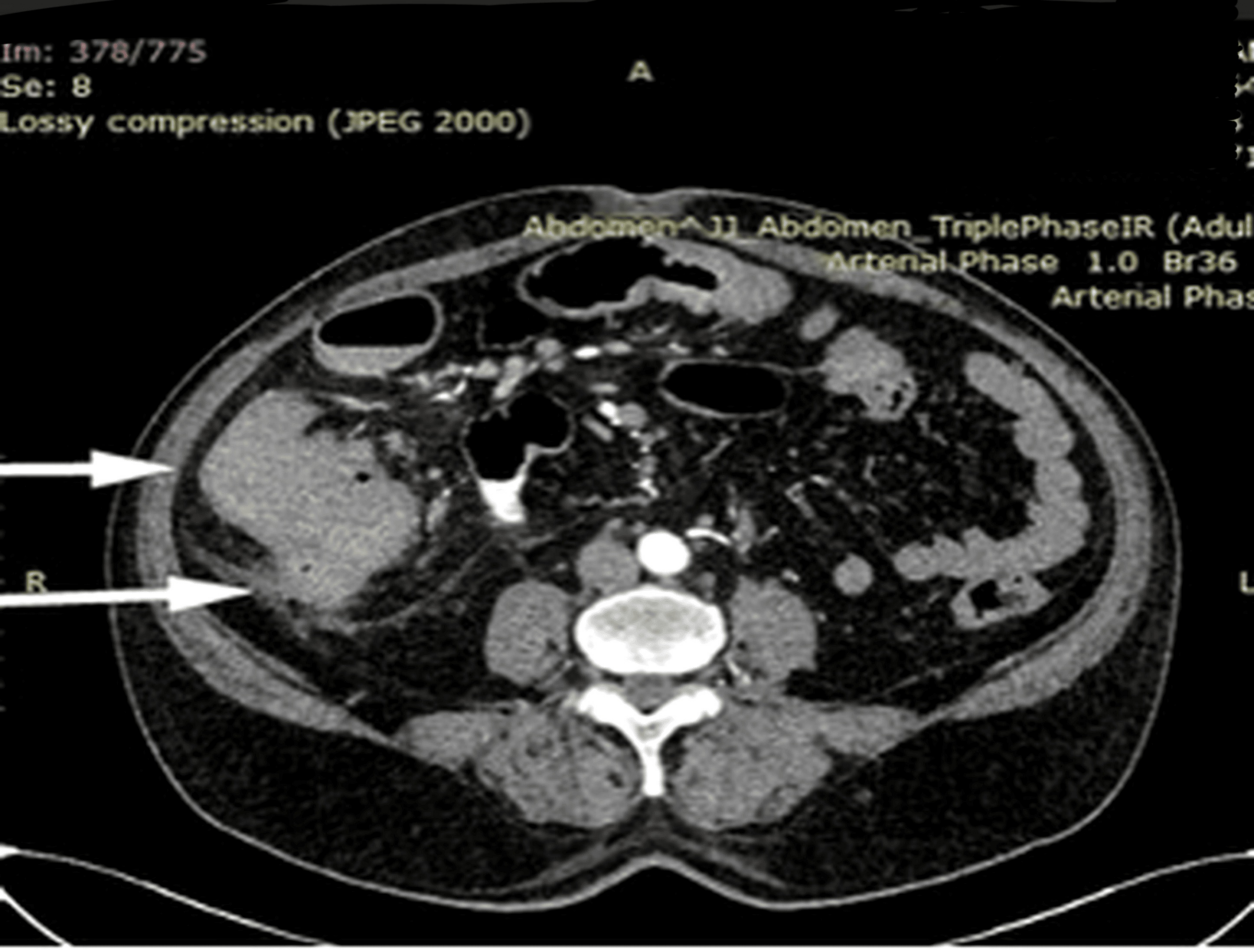
Contrast-enhanced CT abdomen (axial section) showing luminal narrowing suggestive of stricture involving the ascending colon and transverse colon.

One patient presented with a postoperative enterocutaneous fistula following surgery elsewhere, which was successfully managed conservatively by gradual withdrawal of the drain, forming a controlled low-output enterocutaneous fistula eventually leading to spontaneous closure (Figure 7). Surgical intervention was performed in patients who developed complications either during treatment or after completion of ATT, attributable to sequelae of the disease rather than active infection. These complications included stricture formation and fistula formation, commonly presenting with recurrent or subacute intestinal obstruction. Such patients were thoroughly evaluated, nutritionally optimized before surgical intervention. The choice of surgical procedure was individualized based on the extent and nature of the disease. Patients with long-segment or multiple strictures (Figure 8) underwent right hemicolectomy, whereas those with ileal involvement underwent segmental ileal resection or ileocolic resection with primary anastomosis. In cases of single or short-segment strictures, limited resection with primary anastomosis was performed.

Psoas abscess

**Figure 9:**
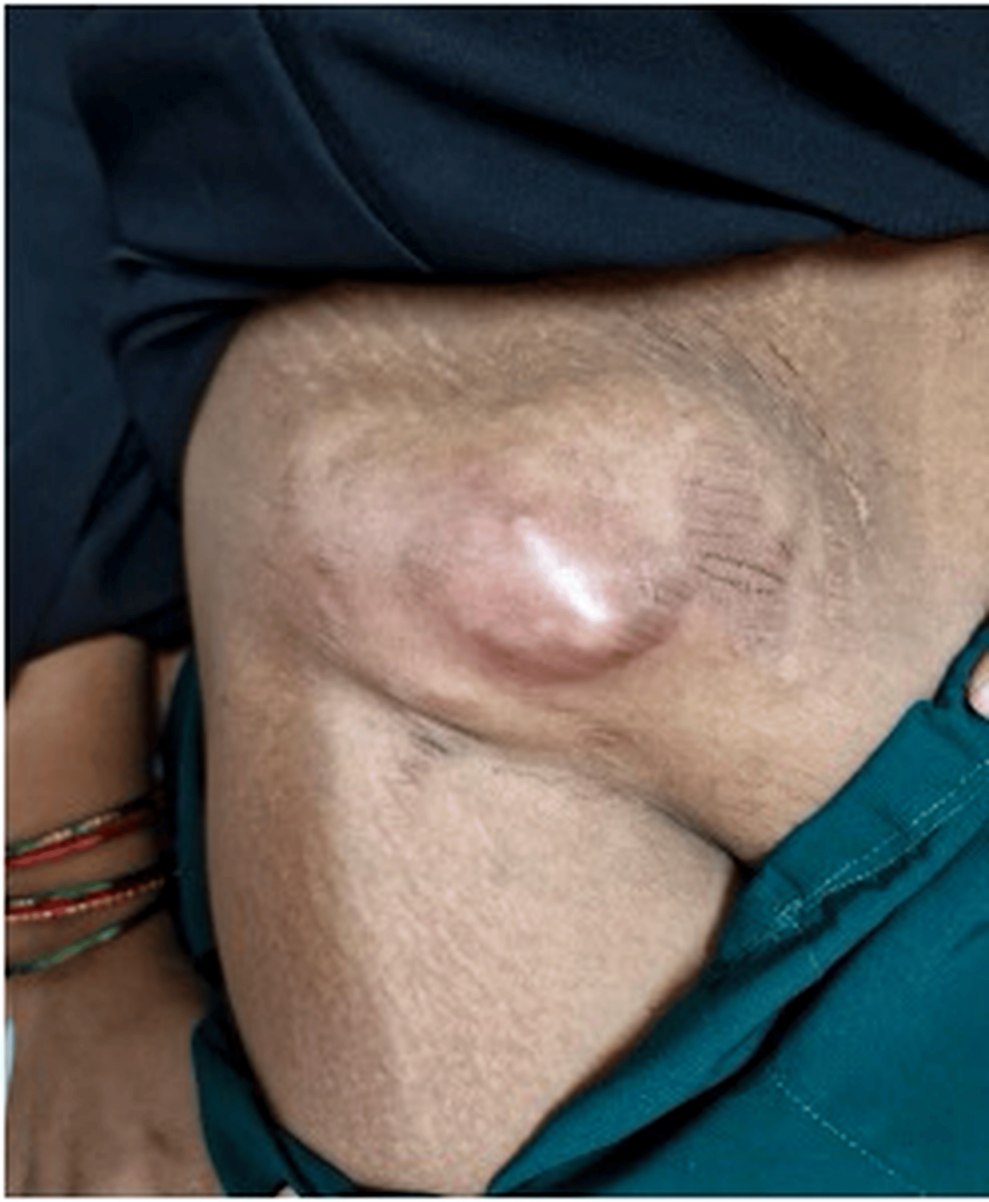
Psoas abscess presenting as abdominal lump.

Among the 26 patients included in the present series, 2 patients were diagnosed with psoas abscess presenting clinically as a RIF swelling (Figure 9). This finding emphasizes that psoas abscess should be considered in the differential diagnosis of patients presenting with RIF swelling, particularly in tuberculosis-endemic regions such as India. In our series, the majority of these cases were secondary psoas abscess of tubercular etiology.

All patients were managed using a conservative, minimally invasive approach. Patients with smaller collections (<60 cc) underwent ultrasound-guided aspiration, whereas those with larger collections (>60 cc) were treated with ultrasound-guided pigtail catheter drainage. In addition, all patients received ATT as per the recommended treatment regimen.

Malignancy

**Figure 10:**
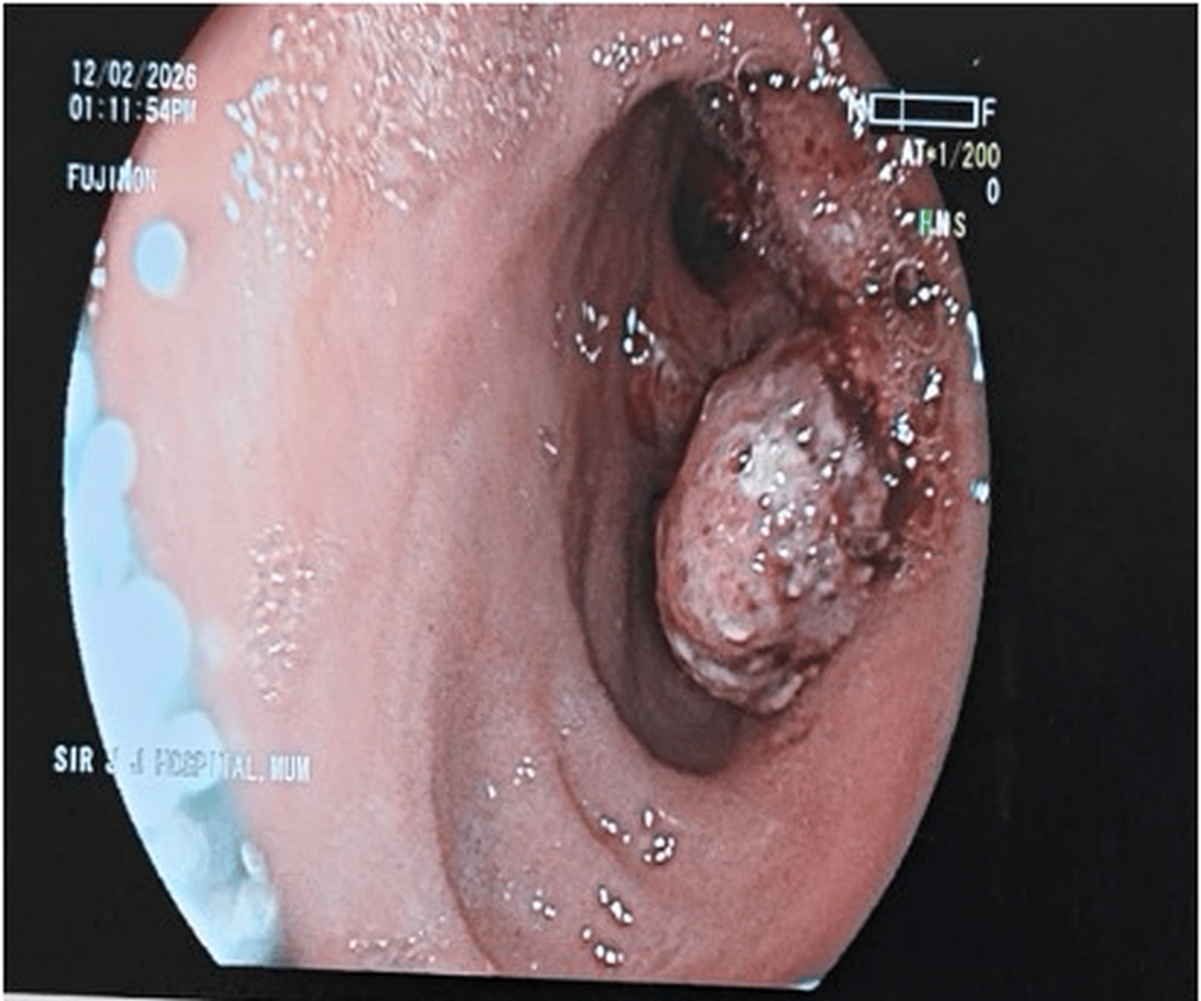
Colonoscopy suggestive of growth

**Figure 11:**
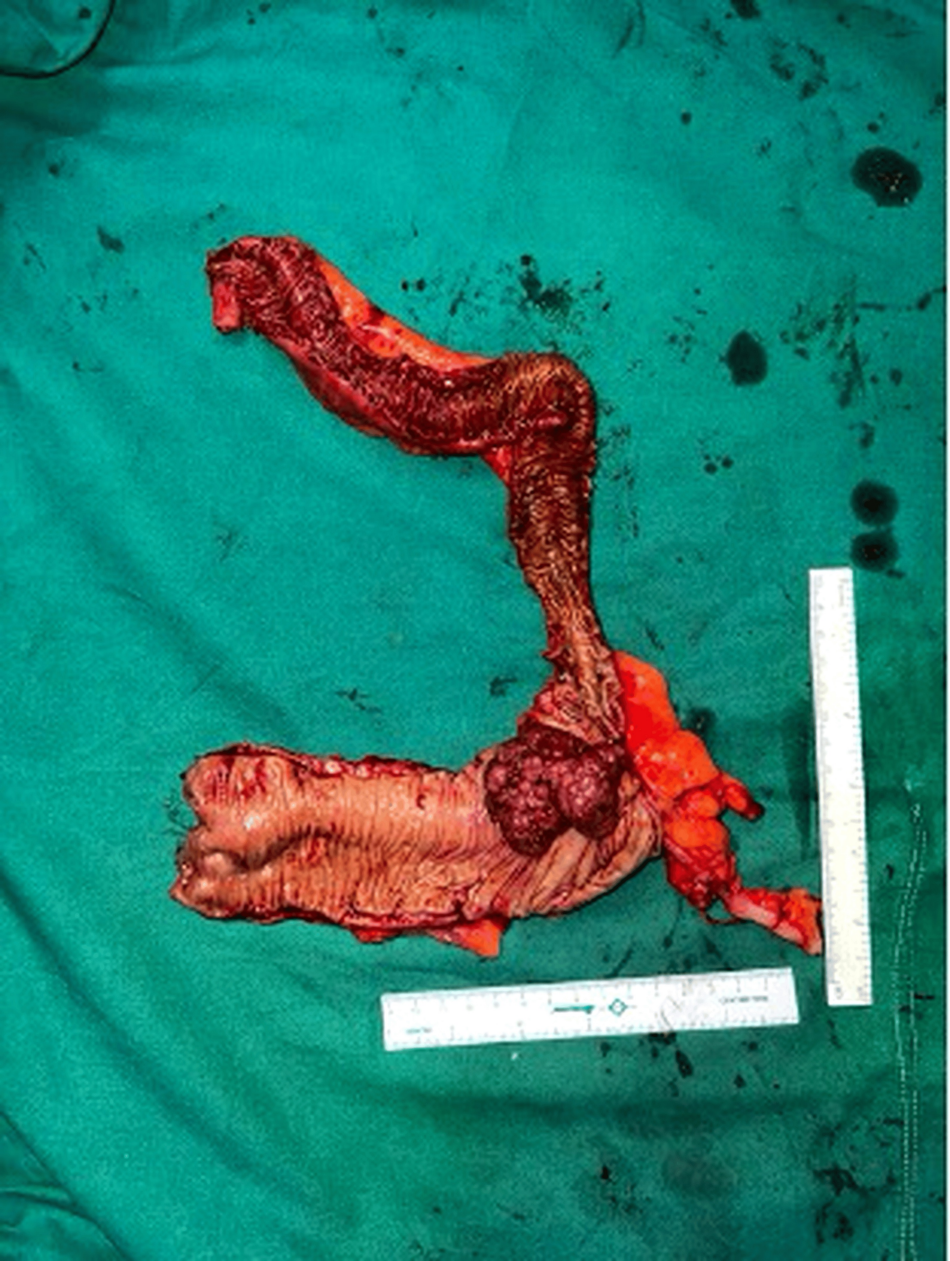
Specimen suggestive of adenocarcinoma of the colon

In this series, two patients presenting with a RIF lump were diagnosed with colonic malignancy. One patient, a 42-year-old female with altered bowel habits and occult blood-positive stools, had imaging suggestive of a cecal mass. Colonoscopy revealed a growth, and biopsy confirmed adenocarcinoma. She subsequently underwent right hemicolectomy, with histopathology confirming cecal adenocarcinoma (Figures 10, 11).

**Figure 12:**
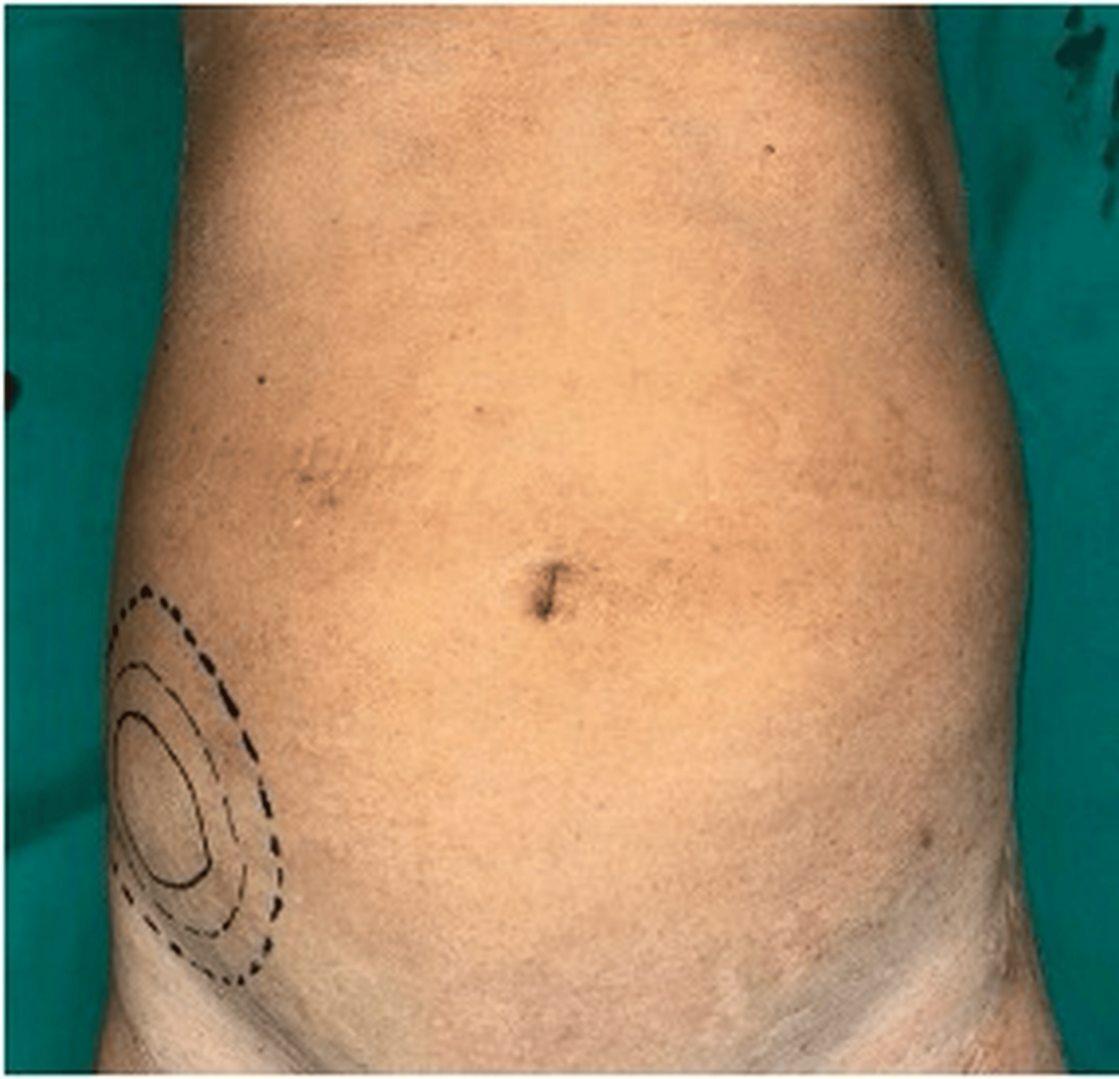
Carcinoma of the colon presenting as a RIF lump RIF, right iliac fossa.

The second patient, a 72-year-old male, presented with a RIF lump, and imaging was suggestive of advanced colonic carcinoma. In view of the locally advanced stage T4b, he was planned for neoadjuvant chemotherapy (Figure 12).

## Discussion

RIF mass represents a common diagnostic dilemma in surgical practice due to its varied etiologies and overlapping clinical features. The most frequent causes include appendicular pathology, ileocecal tuberculosis, psoas abscess, and malignant lesions of the cecum. In the present study, appendicular pathology accounted for the majority of cases, followed by ileocecal tuberculosis, psoas abscess, and malignancy.

Appendicular lump

Appendicular lump, also referred to as appendicular phlegmon, is a localized inflammatory mass formed by the inflamed appendix, omentum, and adjacent bowel loops, typically resulting from delayed or untreated acute appendicitis. Conservative management using the Ochsner-Sherren regimen -- comprising bowel rest, intravenous antibiotics, and close clinical monitoring -- remains the standard initial approach [4]. Previous clinical studies have consistently reported appendicular pathology as the most common cause of RIF mass. Behera et al. demonstrated that appendicular pathology predominated in their series [5]. In our series, comparable findings were observed, with an appendicular lump representing the most common cause of RIF mass. The majority of patients were managed conservatively, and interval appendicectomy was performed selectively in patients who developed recurrent symptoms during follow-up.

Appendicular abscess

Appendicular abscess represents a localized collection of pus surrounding a perforated appendix and is considered an advanced complication of acute appendicitis. Management typically involves intravenous antibiotics with or without image-guided drainage, while surgical intervention is reserved for selected cases. Non-operative management has increasingly been adopted in hemodynamically stable patients. Several studies have evaluated the role of conservative management in appendicular abscess. Andersson and Petzold demonstrated that non-operative management is associated with lower complication rates compared with immediate surgery in patients with appendicular abscess or phlegmon [6]. Similarly, Minaker et al. reported that image-guided percutaneous drainage combined with antibiotics reduces morbidity and hospital stay [7]. In addition, Oliak et al. showed that selective non-operative management with interval appendectomy yields acceptable treatment outcomes in appropriately selected patients [8].

In the present study, seven patients were diagnosed with appendicular abscess and were initially managed conservatively with intravenous antibiotics, with intervention guided by clinical and radiological parameters. Of these, 3 (42.8%) patients developed recurrent symptoms following initial management, requiring readmission and subsequent interval appendectomy, while the remaining 4 (57.2%) patients were managed without the need for surgical intervention. These findings are consistent with existing literature supporting an initial conservative approach. However, the observed recurrence rate of approximately 43% highlights that a substantial proportion of patients may subsequently require delayed surgical intervention. This underscores the importance of careful patient selection, close clinical monitoring, and a stepwise management strategy, wherein conservative treatment is employed initially, followed by interval appendectomy in patients who develop recurrent symptoms.

Ileocecal tuberculosis

Ileocecal tuberculosis is one of the most common forms of abdominal tuberculosis and remains an important cause of RIF mass in endemic regions such as India. Patients typically present with chronic abdominal pain, weight loss, fever, altered bowel habits, or a palpable abdominal lump. Previous studies have consistently reported the ileocecal region as the most frequently involved site in abdominal tuberculosis. Debi et al. reported that the ileocecal region is the most common site of involvement in abdominal tuberculosis due to factors such as physiological stasis and abundant lymphoid tissue [9]. Similarly, Kapoor demonstrated that the majority of patients can be successfully managed with ATT, with surgical intervention required only in cases with complications [10]. In our present study, most patients with ileocecal tuberculosis responded well to ATT, while surgical intervention was required only in selected patients who developed complications such as intestinal obstruction or strictures.

Psoas abscess

Psoas abscess is one of the causes of RIF swelling and may occur as a primary infection or secondary to underlying conditions such as spinal tuberculosis, gastrointestinal infections, Crohn’s disease, or other intra-abdominal inflammatory processes. Patients commonly present with abdominal pain, fever, limp, or restricted hip movements, and the diagnosis is usually established with imaging modalities such as ultrasonography or contrast-enhanced CT, which help determine the location and extent of the abscess. Previous studies have emphasized minimally invasive management for iliopsoas abscess. Rodrigues et al. reported that percutaneous drainage combined with antibiotics is highly effective, with high success rates and reduced need for surgical intervention [11]. Similarly, Dave et al. demonstrated that percutaneous continuous drainage is a safe and effective technique with favorable clinical outcomes and reduced morbidity [12]. In our present study, patients with psoas abscess were managed with image-guided drainage combined with appropriate antibiotic therapy. Collections measuring less than 60 cc were treated with image-guided aspiration, whereas larger collections exceeding 60 cc were managed with pigtail catheter drainage, resulting in satisfactory clinical resolution in most patients.

Malignancy

Carcinoma of the cecum is an important differential diagnosis of RIF mass, particularly in elderly patients, and may present with anemia, weight loss, altered bowel habits, and a palpable abdominal mass. Early diagnosis and surgical resection remain the cornerstone of management. Previous studies have identified carcinoma of the cecum as an important but relatively less frequent cause of RIF mass. Siegel et al. highlighted that colorectal cancer remains a significant global health burden, with right-sided colonic malignancies often presenting late due to non-specific symptoms [13]. Similarly, Benson et al. emphasized that early diagnosis and appropriate surgical management, including right hemicolectomy, significantly improve survival outcomes in patients with colon cancer [14]. In our present study, two malignant cases of cecal carcinoma were identified; one patient with advanced disease was referred for neoadjuvant chemotherapy, while the other underwent surgical management with right hemicolectomy.

**Figure 13:**
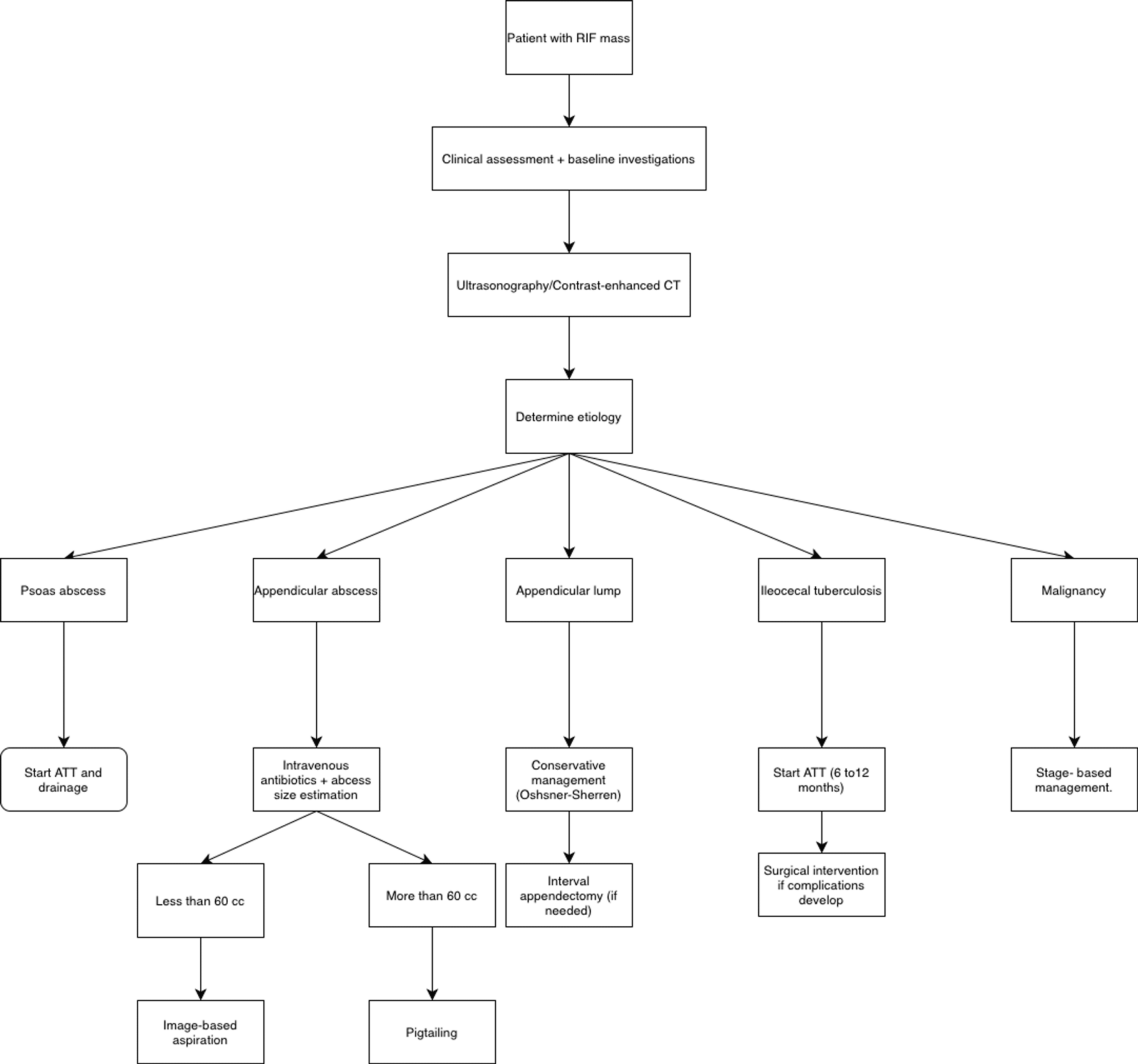
RIF lump management algorithm ATT, anti-tubercular therapy; RIF, right iliac fossa.

The management of RIF mass follows a stepwise, etiology-based approach guided by clinical evaluation and imaging. Appendicular pathologies are primarily managed conservatively, with size-based intervention for abscess and interval appendectomy in selected cases. Ileocecal tuberculosis is treated with ATT, with surgery reserved for complications, while malignancies are managed according to staging. Based on our findings, we propose a practical algorithm (Figure 13) to facilitate timely management of RIF masses, with close monitoring and selective surgical intervention in patients with recurrent or progressive symptoms.

The limitations of the study include its retrospective design, single-center setting, and relatively small sample size. Furthermore, the study population reflects patients managed in a tertiary healthcare center; therefore, the findings may not be directly applicable to rural or non-tertiary healthcare settings. In addition, the lack of long-term follow-up limits the assessment of long-term outcomes and recurrence.

## Conclusions

Appendicular pathology was the most common cause of RIF mass in the present study, with abdominal pain and a palpable lump being the most frequent presenting features. Conservative management was effective in a substantial proportion of cases, particularly in appendicular lump and ileocecal tuberculosis, whereas appendicular abscess and malignancy more often required interventional or surgical management. Early diagnosis through careful clinical evaluation and appropriate imaging is essential in guiding timely and effective management. The proposed algorithm provides a structured, etiology-based approach for standardizing care and improving patient outcomes.
